# Suppression of Inflammatory Immune Responses in Celiac Disease by Experimental Hookworm Infection

**DOI:** 10.1371/journal.pone.0024092

**Published:** 2011-09-16

**Authors:** Henry J. McSorley, Soraya Gaze, James Daveson, Dianne Jones, Robert P. Anderson, Andrew Clouston, Nathalie E. Ruyssers, Richard Speare, James S. McCarthy, Christian R. Engwerda, John Croese, Alex Loukas

**Affiliations:** 1 Queensland Tropical Health Alliance, James Cook University, Cairns and Townsville, Queensland, Australia; 2 Princess Alexandra Hospital, Brisbane, Queensland, Australia; 3 Walter and Eliza Hall Institute, Melbourne, Victoria, Australia; 4 Envoi Specialist Pathologists, Brisbane, Queensland, Australia; 5 Queensland Institute of Medical Research, University of Queensland, Brisbane, Queensland, Australia; 6 The Townsville Hospital, Townsville, Queensland, Australia; Tulane University, United States of America

## Abstract

We present immunological data from two clinical trials where the effect of experimental human hookworm (*Necator americanus*) infection on the pathology of celiac disease was evaluated. We found that basal production of Interferon- (IFN-)γ and Interleukin- (IL-)17A from duodenal biopsy culture was suppressed in hookworm-infected participants compared to uninfected controls. Increased levels of CD4+CD25+Foxp3+ cells in the circulation and mucosa are associated with active celiac disease. We show that this accumulation also occurs during a short-term (1 week) oral gluten challenge, and that hookworm infection suppressed the increase of circulating CD4+CD25+Foxp3+ cells during this challenge period. When duodenal biopsies from hookworm-infected participants were restimulated with the immunodominant gliadin peptide QE65, robust production of IL-2, IFN-γ and IL-17A was detected, even prior to gluten challenge while participants were strictly adhering to a gluten-free diet. Intriguingly, IL-5 was produced only after hookworm infection in response to QE65. Thus we hypothesise that hookworm-induced TH2 and IL-10 cross-regulation of the TH1/TH17 inflammatory response may be responsible for the suppression of these responses during experimental hookworm infection.

## Introduction

Celiac disease is an immune-mediated intestinal inflammatory condition. Uniquely to inflammatory gastrointestinal disease, the stimulatory antigen (dietary gluten) and even the MHC restriction are known [1]. As such, the immunopathology of celiac disease is well understood and allows researchers to focus on the specific population of CD4^+^ T cells that are responsible for inflammation. Predisposition to celiac disease is believed to depend on multiple genetic, immunological and environmental factors [2]. Once the disease is established, however, the processes that lead to pathology are relatively well understood. Dietary gluten is broken down into gliadin in the gut lumen; gliadin is then taken up by the gut epithelium and processed by tissue transglutaminase (tTG) into deamidated gliadin. Expression of tTG is upregulated by the presence of gliadin and in active celiac disease patients produce diagnostic autoantibodies to tTG [3]. Thus, celiac disease is often referred to as an autoimmune disease, as it has autoimmune components, despite the causative agent being “non-self”. Deamidated gliadin peptides bind strongly to HLA-DQ2 or HLA-DQ8 MHC molecules, allowing efficient presentation to CD4+ T cells, and indeed the presence of these MHC haplotypes is the most important genetic risk factor predisposing to disease [4].

Celiac disease has long been thought of as a purely T helper type 1 (TH1) disease, but recent data suggests a role for TH17 cells [5], [6]. Inducing a switch from TH1/TH17 to TH2 has been proposed as a potential treatment for celiac disease [7]. Regulatory T cells (Tregs) also have a role in celiac disease, with recent studies showing increased numbers of circulating and mucosal CD4+Foxp3+ cells in individuals with active celiac disease, as compared to those on a gluten-free diet [8], [9].

Inflammatory gut conditions such as celiac disease are on the rise in the developed world, and one proposed explanation for this is the hygiene hypothesis. This proposes that as populations become more hygienic with a lower incidence of childhood infections (especially parasitic infections), there is a concurrent increase in inappropriate and pathogenic immune responses resulting in increased incidence of allergy, autoimmune and inflammatory gut conditions [10]–[16]. Intervention studies, where populations in developing country settings are treated for parasitic infection using anthelmintic drugs, have also provided evidence for the role of parasites, particularly hookworm [13], [17], [18], in suppression of allergic responses.

Several clinical trials have been conducted using live parasite infections to treat inflammatory bowel diseases and allergies. These include clinical trials using *Trichuris suis* (pig whipworm) to treat Crohn's disease and ulcerative colitis, with reports of resultant remission of symptoms [19]–[21]. Subsequent trials in allergic rhinitis, however, have shown no beneficial effect of *T. suis* [22]. We previously carried out an open label trial using the human hookworm *Necator americanus* in Crohn's disease and observed a trend for reduction in symptom severity [23]. *N. americanus* has also been used in a placebo-controlled clinical trial in an attempt to suppress immunopathology in seasonal asthma however there was no significant benefit in the parasite-infected group compared to the control group [24].

We recently completed a placebo-controlled, double blinded trial exploring the effect of experimental *N. americanus* infection as a treatment for celiac disease [25]. In this trial, 20 subjects, all on a strict gluten-free diet and in remission, were randomly assigned to infection with 15 *N. americanus* larvae (hookworm group) or left uninfected (control). After 20 weeks, both groups were subjected to an oral gluten challenge. Here we present immunological data from this trial (trial 1), and from a continuation of the trial (trial 2) where 7 members of the control group were infected with *N. americanus*, and their responses to a replicate gluten challenge followed. As reported previously [25] no significant reductions in symptom severity were seen in either trial. However, we hypothesised that hookworms suppressed gluten-induced inflammatory immune responses and induced regulatory responses, but this suppression may have been too subtle to lead to significant suppression of pathology in this short-term, small-scale trial. We found that basal TH1 and TH17 responses in the duodenum were suppressed in hookworm-infected participants, and investigated possible mechanisms of immune suppression.

## Materials and Methods

### Clinical Protocol – Trial 1

The design and clinical results of the placebo-controlled clinical trial using hookworm to treat celiac disease have been described elsewhere [25]. This was registered as a clinical trial at ClinicalTrials.gov as NCT00671138. Briefly, twenty otherwise healthy people with HLA-DQ2+ celiac disease on a long-term gluten-free diet were recruited, randomised into 2 groups and either infected percutaneously with 10 infective larvae (L3) of *N. americanus* larvae (“hookworm” group) or given placebo infection using topical chilli pepper (“control” group). Twelve weeks later a boost infection of a further 5 infective larvae (or placebo) was administered. At week 20 post-infection, all subjects were subject to a gluten challenge consisting of four slices of white bread per day for 5 days. This trial will herein be referred to as “trial 1”.

### Clinical Protocol - Trial 2

Approximately 1 year after the completion of trial 1, seven of the ten control subjects participated in a second trial: two subjects could not participate due to other commitments, and one was ineligible due to raised tTG. These 7 participants were infected with *N. americanus*, boosted and challenged with gluten in an identical manner to that described for trial 1. This trial will herein be referred to as “trial 2”.

### Blood and Duodenal Biopsies

To examine the immune response to gluten, duodenal biopsies were taken at weeks 20 (pre-gluten challenge) and 21 (post-gluten challenge) in trial 1, and weeks 0 (pre-infection), 20 and 21 in trial 2. Blood was collected by venepuncture at weeks 0, 20 and 21 in both trials.

### Immunohistochemistry

Duodenal biopsies were fixed in formalin then sectioned for immunohistochemical staining with anti-foxp3 antibodies (eBioscience). Slides were assessed by counting positive cells in a square field of view (FOV), 200 µm by 200 µm. Counts were carried out in a blinded fashion by two independent researchers.

### Acquisition of peripheral blood mononuclear cells

Peripheral blood mononuclear cells (PBMCs) were isolated from heparinised blood over a Ficoll-Paque Plus gradient (GE Healthcare). After separation, PBMCs were counted, and viability was determined by Trypan blue exclusion (>95%). For flow cytometry, PBMCs were frozen in 10% DMSO in fetal calf serum (FCS - Invitrogen); samples from all time-points in each trial were thawed and analysed simultaneously. Cells were stained with anti-CD4-APC, -CD8-PE and -CD25-FITC antibodies (BD Biosciences) then permeabilized and stained with anti-Foxp3-PerCP-Cy5.5 using the Foxp3 kit (Ebioscience) according to manufacturer's protocols. Relevant isotype controls were used for all flow cytometry applications. Flow cytometry was performed using a FACSCalibur or FACSCanto (BD Biosciences).

### Biopsy culture and analysis

For *ex vivo* flow cytometry, four endoscopic pinch biopsies (each approximately 8 mm^3^) from the mid-duodenum were digested for 30 min at room temperature in 0.5 mg/ml collagenase I (Invitrogen), before being passed through a 100 µm nylon filter, yielding 1–2×10^6^ cells. Cells were then stained for CD4, CD8, CD25 and Foxp3 as described above. For biopsy culture, whole biopsies were placed in wells of a 24-well microtiter plate containing 500 µl Tissue Culture Medium (TCM): RPMI-1640 medium (Invitrogen), 10% FCS, 100 U/µl penicillin, 100 µg/ml streptomycin and 2 mM L-glutamine alone or TCM containing 50 µg/ml QE65 peptide (the dominant HLA-DQ2 gliadin peptide [26]), and cultured for 24 h in 95% O_2_/5% CO_2_ at 37°C. Cell-free supernatants were then taken for cytokine bead array (CBA, BD Biosciences) analysis.

### Statistical analysis

All analyses were carried out using Prism 4.0 (Graphpad). When comparing groups and timepoints, where all data was available from both groups (each group n = 10), two-way ANOVA was used with a Bonferroni's post-test to compare timepoints between groups. Where not all data from both groups was available, one-way ANOVA and Bonferroni's post test was used to compare groups and timepoints. When comparing two different timepoints within a group, t tests were used, and where comparing more than 2 timepoints within a group, one-way ANOVA was used. Data was log transformed to normalise distributions where necessary.

## Results

### Cytokine production by duodenal biopsies

**Figure 1:**
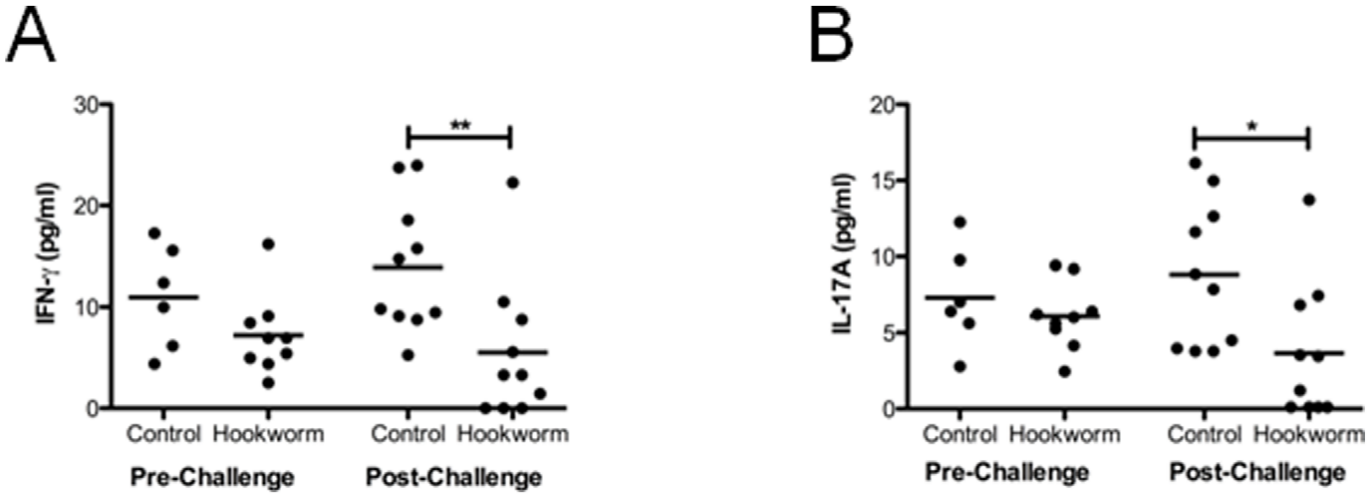
Biopsies taken pre-challenge (week 20) and post-challenge (week 21) from trial 1 and were cultured for 24 hours in tissue culture medium, then supernatants taken for CBA analysis of IFN-γ (A) and IL-17A (B). One-way ANOVA was carried out comparing between groups and timepoints. Unless otherwise indicated differences are not significant. ** = p<0.01, * = p<0.05.

We assessed the inflammatory cytokine response in the duodenum in trial 1 by measuring cytokine levels in supernatants from whole duodenal biopsy cultures taken pre- or post-gluten challenge. Levels of all cytokines assessed were low (<50 pg/ml) as cells were not stimulated *ex vivo*. Levels of the TH1 cytokine IFN-γ and the TH17 cytokine IL-17A tended to increase after gluten challenge in the control group only, but this change did not reach statistical significance. However, levels of IFN-γ and IL-17A were significantly decreased in the hookworm-infected group compared to the control group after gluten challenge (Figure 1A and B).

### Systemic regulatory immune responses

**Figure 2:**
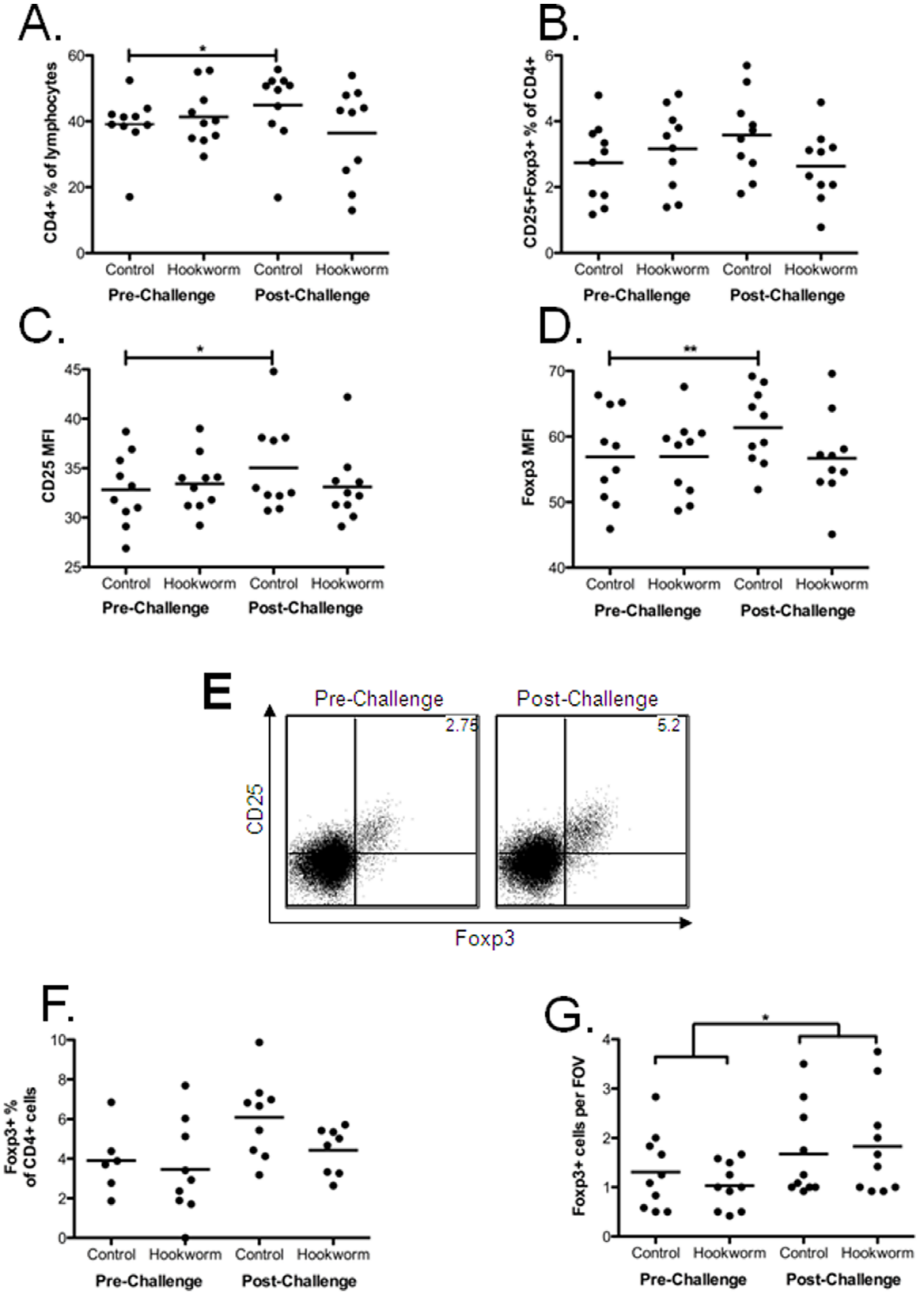
PBMCs from trial 1 pre-challenge (week 20) and post-challenge (week 21) were prepared, cryopreserved and thawed together, then stained for CD4, Foxp3 and CD25. Proportions of CD4^+^ cells in the PBMC lymphocyte population (A). Proportions of CD25^+^Foxp3^+^ cells in CD4^+^ population (B). CD25 MFI (C) and Foxp3 MFI (D), both in the CD4^+^CD25^+^Foxp3^+^ population. Representative Foxp3 versus CD25 plots of cells gated on CD4+ PBMCs are shown in (E). Cell suspensions were prepared from duodenal biopsies pre-challenge and post-challenge, and stained for CD4 and Foxp3. Foxp3+ proportion of the CD4+ population of biopsy cells (F). Formalin fixed duodenal biopsies were also stained for Foxp3 by immunohistochemistry, Foxp3+ cells per high-power field of view (G). Where paired data was available (all except (F)), data were analysed by two-way ANOVA, and if a significant interaction was found paired t tests were used to show differences within groups. For (F), data were analysed by non-parametric ANOVA. N.S. = not significant, * = p<0.05, ** = p<0.01.

Levels of CD4, CD8, CD25 and Foxp3 were assessed in PBMCs taken from participants before and after gluten challenge. After gluten challenge, only the control group showed an increase in the overall proportion of circulating CD4^+^ T cells (control p = 0.018, hookworm p = 0.22) (Figure 2A), a trend for increased proportions of CD25+Foxp3+ CD4^+^ cells (control p = 0.055, hookworm p = 0.24) (Figure 2B) and increased CD25 (Figure 2C) and Foxp3 (Figure 2D) expression (MFI) in the CD4+CD25+Foxp3+ population (p = 0.016 and p = 0.007 respectively), while no change was seen in the hookworm group (p = 0.69 and p = 0.88 respectively). Representative plots are shown in Figure 2E. Consistent with these results, when control individuals were infected with hookworm and re-challenged with gluten in trial 2, no changes were seen in any of the above measurements (CD4+ % of lymphocytes pre-challenge 23.59+/−11.88, post-challenge 23.77+/−7.79 (p = 0.92); CD25+Foxp3+ % of CD4+ pre-challenge 0.65+/−0.25, post-challenge 0.70+/−0.28 (p = 0.47); CD25 MFI in CD4+CD25+Foxp3+ cells pre-challenge 864.4+/−58.3, post-challenge 837.4+/−98.9 (p = 0.25); Foxp3 MFI in CD4+CD25+Foxp3+ cells pre-challenge 5900+/−437.9, post-challenge 6094+/−695.6 (p = 0.34) (mean +/− SD)).

When cells isolated from duodenal biopsies were stained for CD4, CD8, CD25 and Foxp3, a trend for increased proportions of Foxp3+ cells were again seen in the CD4+ population, however in contrast to the PBMC results, this was seen to an equal degree in both groups, and did not reach significance (Figure 2F). In trial 2, however, this increase in proportions of Foxp3+ cells within the CD4+ population reached significance (pre-challenge 1.70+/−0.80, post-challenge 7.74+/−3.37 (p = 0.0014)).. Formalin fixed biopsy sections from trial 1 were stained by immunohistochemistry for Foxp3, confirming flow cytometry data showing that gluten challenge increased numbers of Foxp3+ cells in both groups (Figure 2G).

### Mucosal Immune Response against QE65

**Figure 3:**
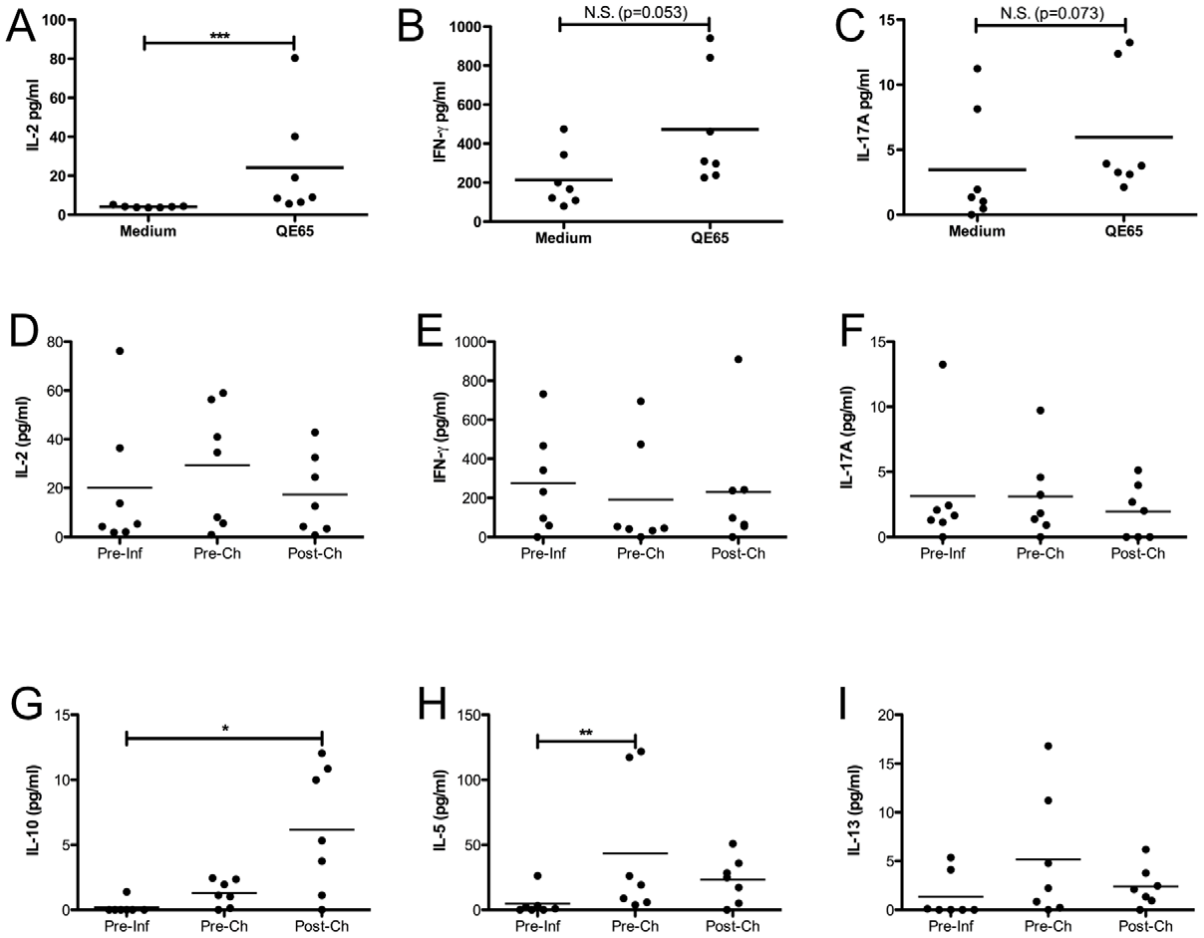
Duodenal biopsies were taken from trial 2 participants pre-infection (Pre-Inf, week 0), pre-challenge (Pre-Ch, week 20) and post-challenge (Post-Ch, week 21) and cultured in either medium alone or with 50 µg/ml QE65 peptide for 24 hours at 37°C in 95% O_2_/5% CO_2_. Supernatants were then taken and levels of cytokines measured by CBA. Results shown in A–C are from the pre-infection timepoint only, showing both medium and QE65 levels. All other results (D–I) have had medium controls subtracted. Cytokines shown are IL-2 (A, D), IFN-γ (B, E), IL-17A (C, F), IL-10 (G), IL-5 (H) and IL-13 (I). A–C was analysed by paired t tests, D–I by 1-way ANOVA. Where data was not normally distributed, log-transformation was carried out and parametric tests used. Differences are not significant unless indicated. * = p<0.05, ** = p<0.01.

To assess the gliadin-specific immune response in the mucosa before (week 0) and after (week 20) hookworm infection and after gluten challenge (week 21), duodenal biopsies from trial 2 were cultured in either medium alone or QE65 peptide, and supernatants were assessed for cytokine production. As shown in Figure 3A, prior to infection or challenge highly significant levels of IL-2 were produced upon stimulation with QE65 compared to medium alone. There was also an apparent IFN-γ and IL-17A response to QE65 although these failed to reach statistical significance (p = 0.053 and p = 0.073 respectively). No significant levels of other cytokines were produced in response to QE65 at this timepoint.

To investigate changes in cytokines produced in response to QE65 over the course of the trial, cytokine levels produced in response to medium alone for each individual were subtracted from those produced in response to QE65, as shown in Figure 3D–I. Levels of QE65-specific IL-2, IFN-γ or IL-17A did not appear to change after hookworm infection or challenge. Production of the regulatory cytokine IL-10 in response to QE65 was significantly increased after gluten challenge, however a small trend for increased levels after challenge, prior to infection, was also seen (Figure 3G). A significant increase in IL-5 produced in response to QE65 was seen after hookworm infection (week 20), with a non-significant trend for increased production of IL-13 (Figure 3H and I). Levels of IL-4 produced following QE65 exposure were below the level of detection (data not shown).

## Discussion

As we recently demonstrated, hookworm administration in celiac disease did not result in a clinically significant suppression of pathology, although a trend towards reduced mucosal inflammation (as assessed by histological scoring) was seen with hookworm infection after gluten challenge [25]. The infective dose of hookworms used in our trial was low (a total of 15 larvae), which although a safe dose [27], may be insufficient to effectively suppress the immunopathogy of celiac disease.

Surprisingly however, we detected suppression of the basal inflammatory immune response in hookworm infected subjects, reflected in the suppression of duodenal IFN-γ and IL-17A production in trial 1. To extend these results, we developed a biopsy restimulation assay for use in trial 2, using the purified QE65 peptide to stimulate gliadin-specific T cells [26]. This allowed us to avoid the use of commercial gliadin preparations containing multiple epitopes, toxic factors and potential endotoxin contamination, which may mask the gliadin-specific T cell response [28]. Using this system we detected robust production of IL-2 to QE65 prior to gluten challenge or infection, and strong trends for production of IFN-γ and IL-17A produced in response to QE65. These results indicate that QE65-specific memory T cells reside in the duodenal mucosa, even after >6 months of apparent strict adherence to a gluten-free diet. QE65-specific responses did not appear to be suppressed by hookworm infection, indicating that QE65-specfic T cell responses may remain high during low intensity hookworm infection, while basal inflammatory cytokine production is suppressed.

As candidate mechanisms for suppression of inflammatory immune responses, we proposed IL-10 production, Treg expansion or activation, and cross-regulation or skewing of the inflammatory immune response to a TH2 response. While we did not detect increased basal IL-10 production by duodenal biopsy cells, we observed IL-10 production after gluten challenge in the hookworm-infected participants in trial 2 in response to QE65; there was also a trend for elevated IL-10 production after hookworm infection but prior to gluten challenge. Therefore hookworm infection may be inducing IL-10, which in turn is suppressing the TH1/TH17 response, however a larger study is required to confirm the existence of a pre-gluten challenge IL-10 response. Of note however, the production of IL-10 (along with IFN-γ) in the duodenum during active celiac disease has been reported elsewhere [29], indicating that the post-challenge IL-10 production may be part of a normal celiac response.

Another candidate mechanism for immunomodulation observed in helminth infections is Treg induction. In mouse models, Tregs are induced by helminth infection [30]–[32], and are responsible for controlling immunopathology [33]. Using Foxp3 as a marker of Tregs, we showed that gluten challenge induces an expansion of mucosal Foxp3+ cells in celiac sufferers irrespective of their hookworm infection status. We also show that in the control, but not the hookworm-infected group, the proportion of CD25+Foxp3+ cells in the circulating CD4+ population increased after gluten challenge, and these cells had increased expression of Foxp3 and CD25. In two recent studies comparing celiac sufferers with active or treated disease, levels of CD4+CD25+Foxp3+ cells in the blood were higher during active disease [8], [9], however this is the first time this effect has been shown during a controlled, short-term gluten challenge. These results, and those from other studies of Tregs in immunopathology [34], [35] and surgical inflammation [36], indicate that CD4+Foxp3+ cells often accumulate during inflammation, however they may not be functionally suppressive [36]. The suppression of effector T cells by regulatory T cells may be rendered unsuccessful due to upregulation of the negative regulator of TGF-β signalling Smad-7 [37]. Therefore counter-intuitively CD4+Foxp3+ cell levels in chronic inflammatory diseases correlate with severity of disease, not resolution. Recent work in Crohn's disease also raises the intriguing possibility that the Foxp3+ cells in the mucosa may also be producing IL-17, and so may not be fully committed to either the TH17 or Treg phenotypes [38]. Unfortunately functional assays of CD4+Foxp3+ suppression were not within the scope of this study, so we cannot say whether the hookworm infection increased suppressive ability and so suppressed inflammatory cytokine production.

We propose that hookworm infection skews the celiac immune response towards a TH2 phenotype, cross-regulating the inflammatory TH1/TH17 response. When duodenal biopsy cultures from trial 2 were restimulated with QE65 peptide, we found no evidence of a TH2 response prior to infection but we did observe significantly increased production of IL-5 (and a trend for increased production of IL-13) to QE65 once the hookworm infection had become patent. This suggests that the anti-gluten response is being skewed away from a TH1/TH17 towards a TH2 phenotype as a consequence of the immune response to hookworm infection. TH2 responses are often associated with IL-10 production, which in turn suppresses inflammatory responses [39]. Evidence for established inflammatory responses being suppressed by TH2 responses comes from coinfection studies, for instance established *Bordetella pertussis*-inducedTH1 responses can be suppressed by *Fasciola hepatica* TH2 responses in an IL-4-dependent manner [40]. Indeed, skewing of the immune response towards TH2 in celiac disease has been suggested previously as a potential immunotherapy [41].

In summary, we present immunological data from a clinical trial using human hookworm to treat celiac disease. We cultured intestinal biopsies in a gliadin-derived peptide and found inflammatory T cell responses could be measured in the absence of *in vivo* gluten challenge, and that even short term gluten challenge increased the levels of CD4+Foxp3+ cells in celiac individuals. Hookworm infection suppressed basal production of the inflammatory cytokines IFN-γ and IL-17A, and our data indicate that this suppression may be dependent upon skewing or cross-regulation of the celiac response by a concurrent TH2 response, and/or IL-10 production. Future efforts should explore the potential protective effect of hookworm on a lesser gluten challenge than that used in this study, addressing the impact of infection on a low gluten diet.
